# Supplementary material for: Administration of Human MSC-Derived Extracellular Vesicles for the Treatment of Primary Sclerosing Cholangitis: Preclinical Data in MDR2 Knockout Mice
Source: Int J Mol Sci. 2020 Nov 23;21(22):8874. doi: 10.3390/ijms21228874 (PMC7700340; doi:10.3390/ijms21228874)
Supplement: Supplementary file 1 [file ijms-21-08874-s001.pdf]

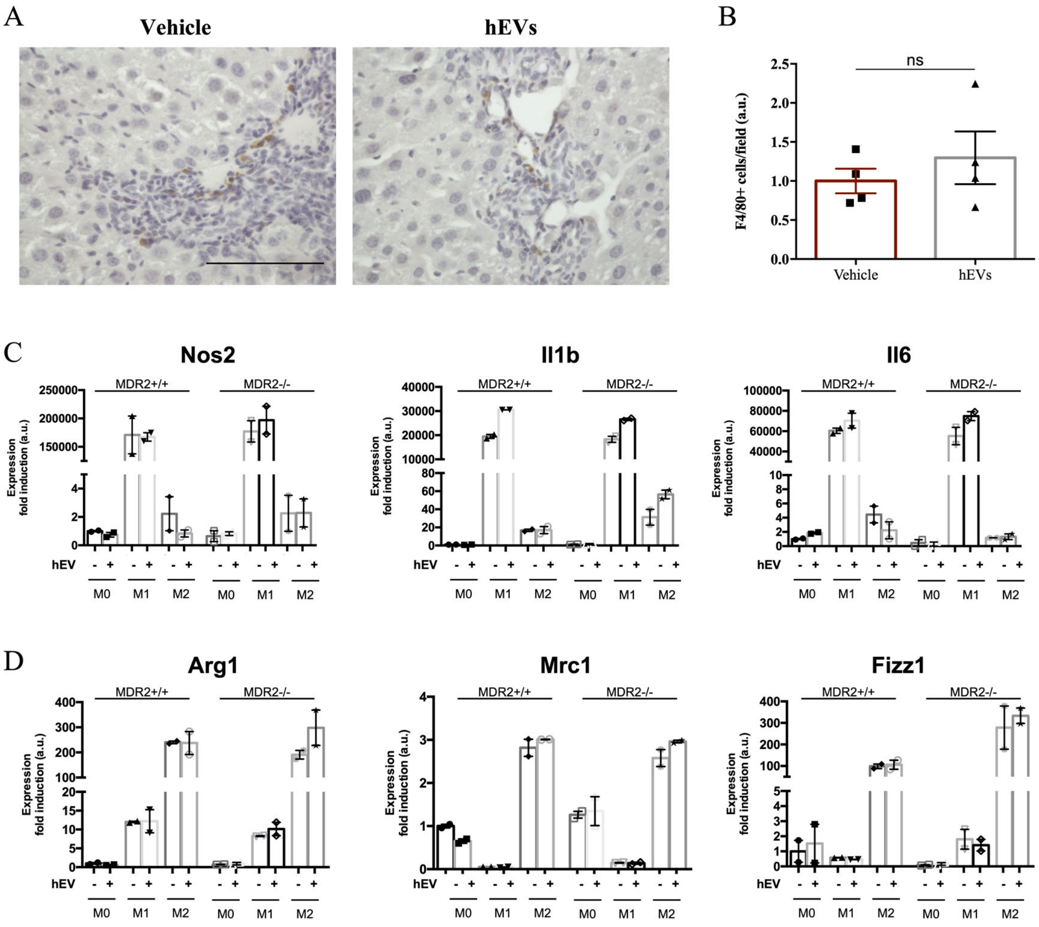

Figure 1. S: (**A**) Representative images of F4/80 immunostaining of liver sections from FVB.Mdr2$^{-/-}$ mice treated or not with EVs. Scale bar, 100 μm (**B**) Relative quantification of F4/80 immunoreactivity of liver section from FVB.Mdr2$^{-/-}$ mice injected with EVs. Bars represent means ± s.e.m from at least 4 sections obtained from 4 animals per group. (**C-D**) Relative mRNA expression of NOS2, Il1b, il6 (**C**), and ARG, MRC and Fizz (**D**) measured by real-time PCR in MDR2$^{-/-}$bone-marrow derived macrophage polarized to M0, M1 or M2 phenotypes either in the presence or absence of hEVs for 24 hours. Bars show expression fold induction normalized on untreated WT M0. Data from two independent experiments, each dot refers to one mouse.
